# Measured cardiorespiratory fitness and self‐reported physical activity: associations with cancer risk and death in a long‐term prospective cohort study

**DOI:** 10.1002/cam4.773

**Published:** 2016-05-26

**Authors:** Trude E. Robsahm, Ragnhild S. Falk, Trond Heir, Leiv Sandvik, Linda Vos, Jan E. Erikssen, Steinar Tretli

**Affiliations:** ^1^ Department of ResearchCancer Registry of NorwayPB 5313 MajorstuenOsloN‐0304Norway; ^2^ Oslo centre for biostatistics and epidemiologyOslo University HospitalPb 4950 NydalenOslo0424Norway; ^3^ Oslo Ischemia studyOslo University HospitalOsloNorway; ^4^ Department of Public Health and General PracticeNorwegian University of Science and TechnologyPB 8905Trondheim7491Norway

**Keywords:** Cardiorespiratory fitness, physical activity, cancer, risk, mortality, fatality

## Abstract

Physical activity is inversely associated with risk of some cancers. The relation with cancer‐specific death remains uncertain. Mainly, studies on relationships between physical activity and cancer are based on self‐reported physical activity (SPA). Hereby, we examined whether measured cardiorespiratory fitness (CRF) is associated with cancer risk, mortality, and case fatality. We also describe relationships between SPA and these outcomes, and between CRF and SPA. A cohort of 1997 healthy Norwegian men, aged 40–59 years at inclusion in 1972–75, was followed throughout 2012. At baseline, CRF was objectively measured. SPA (leisure time and occupational) was obtained through a questionnaire. Relationships between CRF or SPA, and the outcomes were estimated using Cox regression, adjusted for age, body mass index (BMI), and smoking. Pearson correlation coefficients evaluated agreements between CRF and SPA. During follow‐up, 758 men were diagnosed with cancer and 433 cancer deaths occurred. Analyses revealed lower cancer risk (Hazard ratio [HR] 0.85, 95% confidence intervals [CI]: 0.68–1.00), mortality (HR 0.68, 95% CI: 0.53–0.88), and case fatality (HR 0.74, 95% CI: 0.57–0.96), in men with high CRF compared to low CRF. Light leisure time SPA was associated with lower cancer risk (HR 0.70, 95% CI: 0.56–0.86) and mortality (HR 0.64 95% CI: 0.49–0.83), whereas strenuous occupational SPA was associated with higher risks (HR 1.42, 95% CI: 1.13–1.78 and HR 1.45, 95% CI: 1.09–1.93). Correlations between CRF and SPA were 0.351 (*P* < 0.001) and −0.106 (*P* < 0.001) for leisure time and occupational SPA, respectively. A high midlife CRF may be beneficial for cancer risk, cancer mortality, and case fatality.

## Introduction

Over the last decades, a large number of epidemiological studies have investigated whether physical activity is associated with lower cancer risk. In men, however, evidence for such a relationship is only revealed for colon cancer 1, 2. For other cancers, the association is less clear 1, 2. An inverse association is also observed between physical activity and cancer mortality, for total cancer 3, 4, 5, 6, 7, 8, 9 and cancer‐specific for colon, liver, and lung cancer 3, 6, 9, 10. Whether prediagnostic physical activity influences cancer survival in cancer patients is a question given less attention. A positive relationship with survival is, however, indicated in studies of colorectal cancer 11, 12, 13. Physical activity is demonstrated to influence several biological mechanisms (hormonal, immunological, and mechanical) associated with cancer development 14 and, potentially, physical activity may influence development of most major cancers.

The unclear relationship between physical activity and cancer development may be due to difficulties in obtaining reliable data on physical activity habits, particularly over a long time‐span. Self‐reported physical activity (SPA), typically used in epidemiological research, may underestimate the association between physical activity and health outcomes 15, 16. Physical fitness, a set of physiological attributes that are enhanced through regular physical activity, is less prone to misclassification and may better capture health consequences of an active versus sedentary lifestyle than self‐reported activity. Most common in use in health studies is measurement of cardiorespiratory fitness (CRF), which reflects the ability of the body's circulatory and respiratory systems to supply oxygen during sustained physical activity. CRF thus constitutes an objective measure of aerobic activity performed over time 17, and for all‐cause mortality 18 and cardiovascular diseases (CVD) risk and mortality 19, 20 an inverse association with CRF is well established. This provides an expectation of a stronger association between CRF and cancer than found for SPA. CRF assessment, however, is highly resource‐intensive and therefore rarely used in cancer epidemiological research. To our knowledge, few studies have evaluated associations between CRF and cancer risk 21, 22, 23, 24 and case fatality 22, whereas several studies have investigated the association between CRF and cancer mortality in men 23, 25, 26, 27, 28, 29, 30, 31, 32, 33, 34.

Based on a male cohort of initially healthy middle‐aged men, with measurement of CRF and SPA, leisure time and occupational, we aimed to explore whether CRF and SPA were related to overall cancer risk, cancer mortality and cancer case fatality. In addition, we aimed to examine the agreement between CRF and SPA.

## Material and Methods

The study is based on the Oslo Ischemia cohort 35, and data on cancer and death, from the Norwegian Cancer Registry and the Cause of Death Registry, respectively.

### Data sources

*The Oslo Ischemia study* is a comprehensive health survey established in 1972, aimed to examine the prevalence and development of coronary heart disease and other cardiovascular diseases in a healthy male population 35. In total, 2341 healthy employed men, in the age‐group 40–59 years, were invited, of whom 2014 (86%) participated by completing the study protocol. Details about the selection criteria are presented elsewhere 35, 36, 37. At baseline, after 12 h of fasting and 8 h nonsmoking, a comprehensive clinical examination was conducted, including measurements of height, weight, lung capacity, and a panel of blood tests, and a near maximal exercise bicycle test 35. The exercise test was performed step‐wise, with a duration of 6 min on each step, starting at 100 watts. The load was incremented by 50 watts per step 35. In addition, information on lifestyle variables (i.e., smoking habits and physical activity at work and leisure time) was collected through a questionnaire 38. This cohort has not been examined with regard to risks of cancer, cancer mortality, or cancer survival, except for a recent study that found an inverse association between cholesterol and risk of prostate cancer 39.

*The Cancer Registry of Norway* has, since the establishment in 1953, registered data on all malignancies diagnosed in the Norwegian population. Mandatory reporting from several independent sources ensures completeness and high data quality 40. *The Cause of Death Registry* contains information on all recorded deaths of Norwegian citizens living in Norway at time of death since 1st of January 1951. Linkages between the Oslo Ischemia study, the Cancer Registry and the Cause of Death Registry, were possible using the unique 11‐digit personal identification number, which was assigned all Norwegians in 1960 and thereafter to all newborns and persons residing in Norway. The linkages gave complete information on cancer (cancer type, date of diagnosis) vital status, date and cause of death, and date of migration. Permission to link the data was provided by the Regional Committees for Medical and Health Research Ethics.

### Exposure variables

Cardiovascular fitness was measured as total work (sum of work performed in the bicycle test) divided by body weight, kJ/kg, in tertiles, giving the tertile limits: 1: <118.9 (mean 91.9); 2: 119–161.4 (mean 139.1); 3: >161.5 (mean 207.9). Information on SPA, leisure time, and occupational, was extracted from the questionnaire and divided into the following categories: *Leisure time SPA*: no activity, light level (light intensity activity as walking/gardening), moderate/high level (moderate to high intensity activity ≥2 times/week); *Occupational SPA*: sedentary, standing/walking, strenuous. Age at inclusion was divided into four groups (<45, 45–49, 50–54, 55 +  years). Individual body mass index (BMI) was calculated based on the objective measurements of height and weight (body weight/height^2^, kg/m^2^), divided into two categories: low/normal weight (BMI < 25) and overweight/obese (BMI ≥ 25). Based on self‐reported information on smoking, the men were categorized as ever and never smokers.

Of the 2014 men, two were excluded due to missing vital status data and 15 were excluded due to a cancer diagnosis prior to date of the first examination, leaving 1997 men for analyses.

### Statistical analyses

Descriptive analyses were conducted, for the baseline characteristics of the men, presented as means (with ranges), and percentages (%).

**Figure 1:**
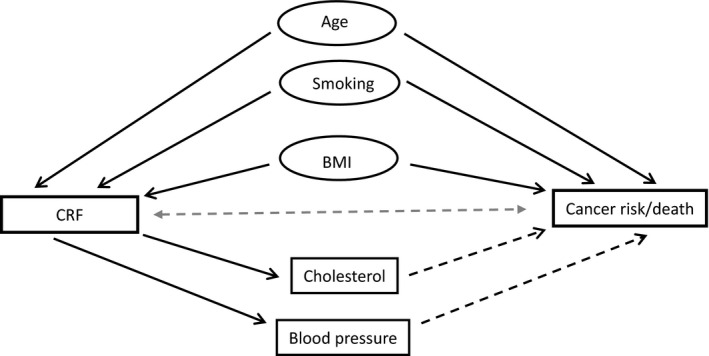
A directed acyclic graph, illustrating whether the available variables in this study are related to cardiorespiratory fitness (CRF) and to cancer risk/death (Solid line = causal relation; dotted line = unclear relation).

Cox regression models were conducted to evaluate the relationship between CRF or SPA (leisure time and occupational) and risks of overall cancer, cancer mortality (cancer as underlying cause of death in the total cohort), and case fatality (cancer as underlying cause of death among those who developed cancer). Hazard ratios (HRs) with 95% confidence intervals (CI) were calculated. The men were followed longitudinally from the date of examination to the date of diagnosis, date of death, emigration, or end of follow‐up, at December 31st 2012. For the estimation of cancer case fatality, cancer cases were followed from the date of diagnosis to date of death, emigration, or end of follow‐up at December 31st 2012. A directed acyclic graph was used to evaluate variables to be included in the regression model (Fig. 1). Potential confounding factors included in final fully adjusted regression model were age, BMI, and smoking.

A corresponding Cox model was used for sensitivity analyses. First, to eliminate the possibility that low CRF or SPA levels result from an ongoing cancer disease (reverse causality), analyses were restricted to men still alive and cancer free 10 years after baseline. Secondly, sensitivity analyses were conducted by restricting the end of follow‐up to the age of 75 years. This was done to examine the possibility of artifactual elevated risks of cancer outcomes in men with high CRF or SPA levels, due to competing cause of death from cardiovascular diseases.

With increasing age, individuals may experience several potential disease endpoints, of which some (i.e., death) prevent the endpoints of interest from occurrence. Therefore, we conducted competing risk analysis, using the Fine and Gray proportional hazard approach 41. The model gives subdistribution hazard ratios (SHRs) with 95% CIs for the relationships between measured CRF or SPA and the cancer outcomes, accounting for competing events. For evaluation of cancer risk, all deaths were considered as competing events, while in evaluation of cancer death, other deaths than those caused by cancer were considered as competing events.

Finally, to examine the agreement between measured CRF and SPA (leisure time and occupational), we calculated mean CRF with 95% CI within each combination of the leisure time and occupational SPA categories. Furthermore, we calculated Pearson's correlation coefficients between CRF and SPA, leisure time, and occupational, respectively. Unadjusted and age, BMI, and smoke‐adjusted correlations were calculated. Adjustments were done using a linear regression model with continuous values for age, BMI and smoke. *P*‐values were calculated using Fisher r to z transformation 42.

All statistical analyses were performed using Stata (Stata statistical software, release 14.0 Collage Station TX, StataCorp LP, 2015).

## Results

Table 1 presents the study cohort characteristics at baseline. Mean time of follow‐up was 26.2 years (95% CI: 25.8–26.7). During follow‐up, 758 men were diagnosed with cancer and 1511 deaths occurred, whereof 433 with cancer as underlying cause.

**Table 1:** Study population characteristics at baseline (*n* = 1997)

Characteristics	Mean	Range	**%**
Age (years)	49.3	37–62	
<45	42.2		23.4
45–49	47.0		28.4
50–54	51.9		27.6
≥55	57.2		20.6
CRF (kJ/kg)	146.2	21.1–553.8	
Tertile 1	91.9	21.1–118.9	
Tertile 2	139.1	119.0–161.4	
Tertile 3	207.9	161.5–553.8	
SPA, occupational			
Sedentary			52.4
Standing/walking			36.0
Strenuous			11.6
SPA, leisure time			
No activity			13.4
Light level			72.1
Moderate/high level			14.5
Weight (kg)	76.8	50.0–122.5	
Height (cm)	176.8	153.5–198.5	
BMI (kg/m^2^)	24.6	17.2–38.9	
<25	22.9	17.2–24.9	60.5
≥25	27.2	25.0–38.9	39.5
Smoking			
Never			25.1
Ever			74.9
Cholesterol (mmol/L)	6.65	2.72–15.41	
Systolic BP (mm/Hg)	130.1	88–220	
Diastolic BP (mm/Hg)	87.1	54–130	

CRF, cardiorespiratory fitness; SPA, self‐reported physical activity; BMI, body mass index; BP, blood pressure.

Compared to the lowest CRF level, men with highest CRF were associated with lower risk of cancer (HR 0.85, 95% CI: 0.68–1.00), cancer mortality (HR 0.68 95% CI: 0.53–0.88), and case fatality (HR 0.74 95% CI: 0.57–0.96) (Table 2). No difference was seen between CRF level 1 and 2, with regard to neither cancer risk, cancer mortality nor case fatality.

**Table 2:** Hazard ratio (HR) and 95% confidence intervals (CIs) for cancer risk, cancer mortality, and cancer case fatality, according to tertiles of cardiorespiratory fitness (CRF), adjusted for age, body mass index, and smoking

	Cancer risk		Cancer mortality		Case fatality	
	Numbers^a^	HR (95% CI)	Numbers^a^	HR (95% CI)	Numbers^a^	HR (95% CI)
**CRF** (kJ/kg) tertiles^b^	1997/758		1997/433		758/433	
1 < 118 (mean 91.9)	667/240	1.00	667/152	1.00	240/152	1.00
2 119–161 (mean 139.1)	665/264	0.98 (0.82,1.17)	665/162	0.98 (0.78,1.23)	264/162	0.93 (0.74,1.16)
3 > 161 (mean 207.9)	665/254	0.85 (0.68,1.00)	665/119	0.68 (0.53,0.88)	254/119	0.74 (0.57,0.96)

a Number; men/failures.

b Tertile limits and means for each tertile.

Sensitivity analyses restricted to men alive and cancer free 10 years after baseline gave similar HRs as found when starting at baseline (Table S1). However, significant differences between low and high CRF were seen only for cancer mortality and case fatality. Analyses restricted to end of follow‐up at age 75 gave correspondingly decreased HRs for high CRF, for all cancer outcomes, as found in the full time analyses (Table S2). However, significant lower HR was only seen for cancer mortality, probably due to less power as a large proportion of the cases (*n* = 332) occurred at ages above 75 years.

Table 3 shows the HRs for cancer risk, cancer mortality and case fatality according to SPA. Compared to men reporting no activity in leisure time, light level SPA was associated with lower cancer risk and mortality (Table 3). No significant association was found for men reporting activity at a moderate/high level. On the other hand, strenuous occupational activity was associated with higher risks of cancer and cancer mortality, compared to sedentary occupations. The HR for case fatality was elevated, although not statistically significant (Table 3).

**Table 3:** Hazard ratio (HR) and 95% confidence intervals (CIs) for cancer risk, cancer mortality, and cancer case fatality, according to self‐reported physical activity (SPA), adjusted for age, body mass index, and smoking

	Cancer risk		Cancer mortality		Case fatality	
	Numbers^a^	HR (95% CI)	Numbers^a^	HR (95% CI)	Numbers^a^	HR (95% CI)
SPA, leisure time^b^						
No activity	268/105	1.00	268/68	1.00	105/68	1.00
Light level	1440/528	0.70 (0.56,0.86)	1440/296	0.64 (0.49,0.83)	528/296	0.80 (0.62,1.05)
Moderate/high level	289/125	0.83 (0.64,1.07)	289/69	0.78 (0.56,1.09)	125/69	0.85 (0.61,1.19)
SPA, occupational						
Sedentary	1047/394	1.00	1047/211	1.00	394/211	1.00
Standing/walking	719/268	1.14 (0.97,1.33)	719/161	1.16 (0.95,1.44)	268/161	1.05 (0.85,1.29)
Strenuous	231/96	1.42 (1.13,1.78)	231/61	1.45 (1.09,1.93)	96/61	1.31 (0.98,1.75)

a Numbers; men/failures.

b No activity (includes no activity reported), light level (occasionally light intensity activity as walking/gardening), moderate/high level (moderate to high intensity activity ≥2 times/week).

The competing risk analyses did not reveal any statistically significant differences between low and high CRF, neither for cancer risk, cancer mortality nor case fatality (Table S3). The associations between SPA and the cancer outcomes taking competing events into account were weaker than those revealed by Cox analyses (Table S4). Only the inverse association between light leisure time SPA and cancer mortality remained statistically significant (SHR 0.76, 95% CI: 0.58–0.99).

Table 4 shows increasing mean CRF by increasing level of leisure time SPA, for each category of occupational activity, whereas mean CRF tended to decrease by increasing occupational SPA, for each level of leisure time activity. A moderate positive correlation was found between CRF and leisure time SPA, whereas a weak negative correlation was found for occupational SPA (Table 5).

**Table 4:** Mean cardiorespiratory fitness (CRF) with 95% confidence interval (CI) for each combination of the self‐reported physical activity (SPA) categories

	Mean CRF (95% CI)		
	SPA, Occupational		
SPA, Leisure time^a^	Sedentary	Standing/walking	Strenuous
No activity	123.2 (112.9–133.4)	113.4 (106.2–120.7)	125.1 (109.6–140.5)
Light level	148.8 (145.3–152.3)	135.5 (131.6–139.3)	129.6 (122.3–136.9)
Moderate/high level	209.0 (196.7–221.3)	182.8 (168.6–197.0)	147.1 (126.5–167.8)

a No activity (includes no activity reported), light level (occasionally light intensity activity as walking/gardening), moderate/high level (moderate to high intensity activity ≥2 times/week).

**Table 5:** Pearson's correlation coefficient (Pr) with *P*‐values (p) between measured cardiorespiratory fitness (CRF) and self‐reported physical activity (SPA), in leisure‐time and occupational

	CRF^a^		CRF^b^	
	Pr	*P*	Pr	*P*
SPA, leisure time	0.345	<0.001	0.351	<0.001
SPA, occupational	−0.160	<0.001	−0.106	<0.001

a Unadjusted.

b Adjusted for age, body mass index, and smoking.

## Discussion

In this study, we found that high CRF was associated with lower cancer risk, cancer mortality and case fatality, compared to men with low CRF. The magnitude of the association found for cancer risk was, however, less than previously reported for specific cancer sites, as colon and lung 22, 23. On the other hand, high CRF has been associated with an increased risk of prostate cancer 21, 22, 23, which also recently was found in a study based on the present cohort 39. If individuals with high CRF are more concerned about health and, thus, more likely to test for prostate cancer, than individuals with low CRF, the positive relation may result from differences in diagnostic intensity. Norway has, however, no cancer screening programs for men, although opportunistic screening for prostate cancer, by PSA‐testing, has been practiced since early 1990s. Prostate, colon, and lung cancers are the most common cancers in Norwegian men, accounting for 50% of all cancer cases 43. If the direction of relations between CRF and cancer differ by cancer site, this may explain that the magnitude of association found for total cancer risk was moderate.

The association found between high CRF and cancer mortality, corresponds well with what reported in previous studies 23, 25, 26, 27, 28, 29, 30, 31, 33, 34. In a recently published meta‐analysis, based on 71,651 individuals and 2002 cancer deaths, they demonstrate an evident dose–response relationship between CRF and cancer mortality 32.

To our knowledge, the only study reporting estimates for the relationship between CRF and cancer case fatality is the study by Lakoski et al. 22. They found a significant association between high midlife CRF and survival after a cancer diagnosis. Our result is in accordance with their finding, indicating that prediagnostic CRF may be of importance for prognosis after a cancer diagnosis.

To reduce the possibility that an inverse relationship between CRF and cancer outcomes resulted from poor CRF test results, due to an ongoing cancer disease, we restricted analyses to men still alive and cancer free 10 years after baseline. The analyses, however, did not confirm such an explanation. In all, our findings, together with those previously reported support a beneficial role of CRF in cancer development.

For leisure time SPA, we found associations between a light activity level and lower risk of cancer and cancer mortality. No relationship was seen for case fatality. Our results correspond well with a study by Kampert et al, based on a large US cohort 27. As in this study, they had information on CRF and SPA, but investigated the associations with cancer mortality only. Kampert et al. found that high CRF was associated with lower cancer mortality, whereas for SPA, a moderate activity level was associated with lower risk 27. The findings indicate that light activity may be of importance, although it may not be correctly reflected in a CRF test. The modest correlation we found between CRF and SPA leisure time (0.351, *P* < 0.001) may indicate that the activity captured with self‐report could be too broad. The correlations we observed concurs with findings in previous studies 16, 44 and support the assumption that difficulties of obtaining reliable data on physical activity may influence the relationship with cancer.

Furthermore, we found positive associations between strenuous occupational SPA and risk of cancer and cancer mortality, and a negative correlation between CRF and occupational SPA. A possible explanation for may be that men who hold sedentary and strenuous occupations represent contradictory socioeconomic levels, high and low, respectively, reflecting differences in important lifestyle factors and health concern that are differently related to cancer 45. Unfortunately, we lack information on socioeconomic variables (i.e., education, income), and were thus not able to take such variables into account in the analyses. Few studies, however, have found a protective role of occupational activity, but most studies that report inverse association between cancer and physical activity are based on leisure lime SPA 46. A reason for this may be that most studies are undertaken in high‐income countries, where leisure time activity accounts for a greater proportion of the total physical activity dose 1.

In the present cohort, with a long time of follow‐up, the most common and competing event was death from CVD 36, 37. Although both CRF and SPA and the covariates age, BMI, and smoking are related to CVD death, the subhazard ratios, calculated by the Fine and Gray model 41, should reflect the influence of the covariates isolated to the events of interest (cancer outcomes). Nevertheless, men in the highest CRF level may have a prolonged lifetime due to prevention of CVD death 36, 37, 47. Consequently, competing risk analysis may result in an artifactual elevated risk of cancer development and a weakening of the potentially inverse relationships between CRF and cancer outcomes. Therefore, the Cox model seems most appropriate to answer our etiological questions, censoring competing events throughout follow‐up instead of incorporating time to both endpoints in the same model 48. However, to eliminate the possibility that prolonged survival in men with high CRF, due to less CVD, influenced the associations to cancer, Cox analyses were restricted to end at age 75. Although having less power, this analysis gave results in line with results from the full time Cox analyses.

On strength of our study is complete information on objectively measured CRF in 1997 men. Furthermore, we were able to follow the cohort prospectively for cancer outcomes over a 40‐year period, with complete and valid information on cancer diagnoses and cause of death during the time‐span covered. A strength is also the individual‐level information on several potential confounding variables. Lastly, the cohort of men has been shown to be representative for their age‐group of men, with regard to cancer occurrence in the counties the men were recruited from (Oslo and Akershus), at this time period 39. Limitations of importance are the size of the cohort, male sex only, and assessments of variables at baseline, making us unable to account for changes over the life course. Furthermore, the questions used for measurement of SPA has not been validated according to current criteria for validation. However, we will underline that the health survey the study is based on took place in the early 1970s, when validation of questions was less usual. The questionnaire included simple questions about physical activity, representative of the time, at work and in leisure time. The aspects of frequency, intensity, and regularity, however, were taken into account in predefined replay options.

## Conclusion

In this 40‐year follow‐up of initially healthy men, a high midlife CRF was associated with a reduced risk of cancer and cancer death, indicating a beneficial role of high CRF in cancer development. CRF was modestly correlated with leisure time SPS, and negatively correlated with occupational SPA. The findings for SPA, indicate that information on physical activity based on self‐report may be biased. More large‐scale cohort studies and surveillance systems including measured CRF are required to reveal the role of physical activity in cancer development.

## Conflict Of Interest

None declared.

## Supporting information


**Table S1.** Hazard ratio (HR) and 95% confidence intervals (CIs) for cancer risk, cancer mortality, and cancer case fatality, according to tertiles of cardiorespiratory fitness (CRF), restricting start of follow‐up to 10 years after baseline, adjusted for age, body mass index, and smoking.
**Table S2**. Hazard ratio (HR) and 95% confidence intervals (CIs) for cancer risk, cancer mortality, and cancer case fatality, according to tertiles of cardiorespiratory fitness (CRF), restricting end of follow‐up to age of 75 years, adjusted for age, body mass index, and smoking.
**Table S3.** Subdistribution hazard ratio (SHR) and 95% confidence intervals (CIs) for cancer risk, cancer mortality, and cancer case fatality, according to tertiles of cardiorespiratory fitness (CRF), adjusted for age, body mass index, and smoking.
**Table S4.** Subdistribution hazard ratio (SHR) and 95% confidence intervals (CIs) for cancer risk, cancer mortality, and cancer case fatality, according to self‐reported physical activity (SPA), adjusted for age, body mass index, and smoking.
