# Supplementary material for: Measured cardiorespiratory fitness and self‐reported physical activity: associations with cancer risk and death in a long‐term prospective cohort study
Source: Cancer Med. 2016 May 26;5(8):2136–44. doi: 10.1002/cam4.773 (PMC4884631; doi:10.1002/cam4.773)
Supplement: Supplementary file 1 — Table S1. Hazard ratio (HR) and 95% confidence intervals (CIs) for cancer risk, cancer mortality, and cancer case fatality, according to tertiles of cardiorespiratory fitness (CRF), restricting start of follow‐up to 10 years after baseline, adjusted for age, body mass index, and smoking. Table S2. Hazard ratio (HR) and 95% confidence intervals (CIs) for cancer risk, cancer mortality, and cancer case fatality, according to tertiles of cardiorespiratory fitness (CRF), restricting end of follow‐up to age of 75 years, adjusted for age, body mass index, and smoking. Table S3. Subdistribution hazard ratio (SHR) and 95% confidence intervals (CIs) for cancer risk, cancer mortality, and cancer case fatality, according to tertiles of cardiorespiratory fitness (CRF), adjusted for age, body mass index, and smoking. Table S4. Subdistribution hazard ratio (SHR) and 95% confidence intervals (CIs) for cancer risk, cancer mortality, and cancer case fatality, according to self‐reported physical activity (SPA), adjusted for age, body mass index, and smoking. [file CAM4-5-2136-s001.docx]

**SUPPLEMENTARY TABLES: S1-S4**

**Table S1.** Hazard ratio (HR) and 95% confidence intervals (CIs) for cancer risk, cancer mortality and cancer case fatality, according to tertiles of cardiorespiratory fitness (CRF), restricting start of follow-up to 10 years after baseline, adjusted for age, body mass index and smoking.

|  | ***Cancer risk*** | | ***Cancer mortality*** | | ***Case fatality*** | |
| --- | --- | --- | --- | --- | --- | --- |
|  | ***Numbers^1^*** | ***HR (95% CI)*** | ***Numbers^1^*** | ***HR (95% CI)*** | ***Numbers^1^*** | ***HR (95% CI)*** |
| **CRF** (kJ/kg),tertiles***^2^***  1 <118 (mean 91.9)  2 119-161 (mean 139.1)  3 >161 (mean 207.9) | 575/201  616/235  639/241 | 1.00  1.00 (0.83,1.22)  0.88 (0.71,1.07) | 575/124  616/141  639/111 | 1.00  0.98 (0.77,1.25)  0.72 (0.56,0.94) | 201/124  235/141  241/111 | 1.00  0.93 (0.74,1.19)  0.70 (0.53,0.91) |

***^1^***Numbers; men at risk/failure

***^2^***Tertile limits and means for each tertile

**Table S2**. Hazard ratio (HR) and 95% confidence intervals (CIs) for cancer risk, cancer mortality and cancer-specific death, according to tertiles of cardiorespiratory fitness (CRF), restricting end of follow-up to age of 75 years, adjusted for age, body mass index and smoking.

|  | ***Cancer risk*** | | ***Cancer mortality*** | | ***Case fatality*** | |
| --- | --- | --- | --- | --- | --- | --- |
|  | ***Numbers^1^*** | ***HR (95% CI)*** | ***Numbers^1^*** | ***HR (95% CI)*** | ***Numbers^1^*** | ***HR (95% CI)*** |
| **CRF** (kJ/kg), tertiles***^2^***  1 <118 (mean 91.9)  2 119-161 (mean 139.1)  3 >161 (mean 207.9) | 667/130  665/154  665/142 | 1.00  1.03 (0.81,1.30)  0.84 (0.65,1.09) | 667/62  665/71  665/45 | 1.00  1.06 (0.75,1.51)  0.66 (0.44,0.99) | 130/62  154/71  142/45 | 1.00  1.00 (0.71,1.42)  0.72 (0.47,1.08) |

***^1^***Numbers; men at risk/failure

***^2^***Tertile limits and means for each tertile

**Table S3.** Sub-distribution hazard ratio (SHR) and 95% confidence intervals (CIs) for cancer risk, cancer mortality and cancer case fatality, according to tertiles of cardiorespiratory fitness (CRF), adjusted for age, body mass index and smoking.

|  | ***Cancer risk*** | ***Cancer mortality*** | ***Case fatality*** |
| --- | --- | --- | --- |
|  | ***SHR (95% CI)*** | ***SHR (95% CI)*** | ***SHR (95% CI)*** |
| **CRF** (kJ/kg), tertiles***^1^***  1 <118 (mean 91.9)  2 119-161 (mean 139.1)  3 >161 (mean 207.9) | 1.00  1.14 (0.95,1.37)  1.10 (0.91,1.33) | 1.00  1.16 (0.92,1.46)  0.96 (0.74,1.23) | 1.00  0.96 (0.76,1.21)  0.78 (0.60,1.02) |

***^1^***Tertile limits and means for each tertile

**Table S4.** Sub-distribution hazard ratio (SHR) and 95% confidence intervals (CIs) for cancer risk, cancer mortality and cancer case fatality, according to self-reported physical activity (SPA), adjusted for age, body mass index and smoking.

|  | ***Cancer risk*** | ***Cancer mortality*** | ***Case fatality*** |
| --- | --- | --- | --- |
|  | ***HR (95% CI)*** | ***HR (95% CI)*** | ***HR (95% CI)*** |
| **SPA, leisure time*^1^***  No activity  Light level  Moderate/high level | 1.00  0.87 (0.70,1.08)  1.09 (0.83,1.42) | 1.00  0.76 (0.58,0.99)  0.96 (0.68,1.34) | 1.00  0.81 (0.63,1.06)  0.84 (0.60,1.17) |
| **SPA, occupational** Sedentary  Standing/walking  Strenuous | 1.00  0.99 (0.85,1.17)  1.14 (0.91,1.45) | 1.00  1.05 (0.85,1.29)  1.21 (0.90,1.63) | 1.00  1.04 (0.86,1.29)  1.21 (0.89,1.64) |

***^1^***No activity (no activity reported), light level (occasionally light intensity activity as walking/gardening), moderate/high level (moderate to high intensity activity ≥2 times/week)
